# The Modulatory Role of Bioactive Compounds in Functional Foods on Inflammation and Metabolic Pathways in Chronic Diseases

**DOI:** 10.3390/foods14050821

**Published:** 2025-02-27

**Authors:** Zheng Feei Ma, Caili Fu, Yeong Yeh Lee

**Affiliations:** 1 Centre for Public Health and Wellbeing, School of Health and Social Wellbeing, College of Health, Science and Society, University of the West of England, Bristol BS16 1QY, UK; 2 Jiangsu Co-Innovation Center of Efficient Processing and Utilization of Forest Resources, College of Chemical Engineering, Nanjing Forestry University, Nanjing 210037, China; 3 School of Medical Sciences, Universiti Sains Malaysia, Kota Bharu 15200, Malaysia

**Keywords:** health promoting, non-communicable diseases, immune response, cytokines, metabolism

## Abstract

Chronic diseases are major contributors to global morbidity and mortality. More than 70% of deaths worldwide are caused by chronic diseases, including cardiovascular diseases (CVDs), obesity, type 2 diabetes, and cancer. These diseases are characterised by chronic low-grade inflammation and metabolic dysregulation. Incorporating functional foods into daily diet has been suggested as a complementary strategy to promote health and lower the risk of non-communicable diseases. Functional foods, known as foods that confer health benefits beyond basic nutrition, have been reported to exhibit preventive and therapeutic benefits such as anti-inflammatory properties for human health. Therefore, the aim of this state-of-the-art review will synthesise the findings from recent and high-quality studies that investigated the modulatory role of some commonly reported bioactive active compounds, such as polyphenols, omega-3 fatty acids, probiotics, and prebiotics, in inflammation and metabolic pathways.

## 1. Introduction

More than 70% of deaths worldwide are caused by chronic diseases, including cardiovascular diseases (CVDs), obesity, type 2 diabetes, and cancer [1]. A key feature of these chronic diseases is persistent low-grade inflammation, which disrupts metabolic processes [2]. Inflammation is a natural defence mechanism that helps the body respond to injury and infections, aiming to restore balance [2]. When tissues are damaged, both the innate and adaptive immune systems become active, triggering the release of various inflammatory substances including eicosanoids, vasoactive amines, cytokines, chemokines, and products of proteolytic cascades [3].

Incorporating functional foods into daily diet has the potential to serve as a complementary strategy for disease prevention. However, various terms have been used to describe foods that support health and help prevent disease, often with overlapping meanings. For example, the term “designer foods” was introduced in 1989 to refer to foods that either naturally contain or are fortified with bioactive non-nutritive plant compounds thought to lower cancer risk [4]. In the same year, the U.S. Foundation for Innovation in Medicine coined the term nutraceuticals, defining them as “any substance that is a food or a part of a food and provides medical or health benefits, including the prevention and treatment of disease” [5]. However, this broad definition lacks regulatory clarity, encompassing a wide range of substances without clear scientific distinction [5]. In contrast, Japan introduced the concept of functional foods for Foods for Specified Health Use (FOSHU) in the mid-1980s. FOSHU is defined as “foods which are expected to have certain health benefits, and have been licensed to bear a label claiming that a person using them for specified health use may expect to obtain the health use through the consumption thereof” by the Japanese Ministry of Health, Labour and Welfare. Unlike designer foods and nutraceuticals, FOSHU requires scientific evidence and official approval before making health claims [6]. During the late 1990s, the European Commission launched the Functional Food Science in Europe (FuFoSE) initiative to establish a scientific framework for evaluating these foods. According to the FuFoSE, a food product is considered functional only if it provides health benefits beyond basic nutrition by positively influencing one or more physiological functions. These benefits may include enhancing overall well-being, improving physical health, or reducing the risk of disease progression [7].

Based on the above definitions, a functional food can take various forms, including a natural unmodified food with inherent health benefits or one that has been altered to enhance its functionality [8] (Table 1). This modification may involve enhancing a specific component through specialised growing conditions or biotechnology [8]. Functional foods can also be fortified with beneficial compounds, modified to remove undesirable components through technological or biotechnological processes or contain a substituted ingredient with improved health properties. Additionally, functional foods may undergo enzymatic, chemical, or technological modifications to enhance their beneficial effects or improve the bioavailability of key nutrients [9].

Given the global rise in non-communicable diseases, leveraging the benefits of functional foods may offer sustainable and effective solutions for improving public health outcomes. However, most studies have investigated the effects of specific nutrients in functional foods administered at high doses on chronic diseases, which may not be relevant to human consumption. Therefore, in this state-of-the-art review, we will synthesise findings from recent studies on the modulatory effects of common bioactive compounds including polyphenols, omega-3 fatty acids, probiotics, and prebiotics in relation to inflammation and metabolic pathways.

## 2. Search Methodology

To examine the current scientific evidence on the modulatory role of functional foods in modulating inflammation and metabolic pathways, a comprehensive literature search was performed using the PubMed, Scopus, and Web of Science databases. The search included studies published up to 2025, identified through keyword combinations such as “functional foods”, “inflammation”, “metabolism”, “chronic disease”, and “non-communicable diseases”. To ensure the inclusion of high-quality and relevant research, only peer-reviewed original studies were considered, while editorials, commentaries, and conference abstracts lacking primary data were excluded.

## 3. Epidemiological Evidence on Functional Foods and Chronic Diseases

By 2030, non-communicable diseases (NCDs) are expected to be responsible for over 75% of deaths worldwide, and the global economic burden of chronic diseases is projected to reach USD 47 trillion [10]. Epidemiological and experimental research studies have consistently reported that a diet rich in vegetables, fruits, spices, fish, legumes, whole grains, and beverages, along with a high fibre intake, can significantly reduce the risk of chronic diseases [11,12]. These include cancer, diabetes, CVD, Alzheimer’s disease, and age-related functional decline. The protective effects are largely attributed to the bioactive compounds found in these foods, which exert anti-inflammatory, antioxidant, and other beneficial physiological effects (Table 2) [13,14,15,16,17,18,19,20,21,22,23,24,25,26,27,28,29,30].

## 4. Polyphenol-Rich Foods

Over the past decade, there has been increasing interest in polyphenol-rich foods due to their potential role in mitigating oxidative stress through antioxidant activity [7]. Polyphenols, naturally occurring compounds in plants, are abundant in vegetables, nuts, fruits, and other plant-based foods [11,12,13]. Their antioxidant properties primarily stem from their ability to neutralise free radicals. In plants, polyphenols serve as a defence mechanism against ultraviolet radiation and pathogens. Based on the number of phenol rings in their structure and the specific linkages between these rings, polyphenols are categorised into several classes [11]. For example, phenolic acids, flavonoids, lignans, and stilbenes represent the four main categories of polyphenols (Table 3).

Numerous in vivo and in vitro studies have been conducted to assess the health effects of polyphenols [14,15,16,17,18,19,20,21,22,23,24,25,26,27,28,29,30]. These polyphenol compounds play a crucial role in protecting the body against external stressors and in neutralising reactive oxygen species (ROS) towards the prevention of oxidative stress-related pathological diseases [15]. Found abundantly in tea, chocolate, fruits, and vegetables, polyphenols have demonstrated the potential to support human health through their antioxidant, anti-inflammatory, and other bioactive properties [15]. For example, the consumption of dark chocolate, which is associated with the intake of flavan-3-ols, including epicatechin, has been associated with a decreased risk of type 2 diabetes [16]. Also, cocoa flavan-3-ols have been reported to lower the risk of stroke, metabolic syndrome, osteoporosis, and myocardial infarction [17,18,19]. Additionally, dietary polyphenols contribute to improved lipid profiles, insulin sensitivity, reduced systemic inflammation, and blood pressure regulation [20].

Phenolic acids, including chlorogenic acids found in coffee, play a significant role in diet, with evidence suggesting that regular coffee consumption is linked to a reduced risk of osteoporosis, type 2 diabetes, and dementia [21,22,23]. Moreover, chlorogenic acid has demonstrated in vivo prebiotic properties, contributing to the prevention of obesity, metabolic and lifestyle-related diseases such as hypertension, and insulin resistance via the AMPKα-LXRα/SrebP-1C pathway [24]. In addition, some epidemiological studies reported an inverse relationship of higher intakes of ferulic acid and caffeic acid with a lower risk of prostate cancer [25,26,27].

Stilbenes are plant-derived secondary metabolites produced through the phenylpropanoid pathway and play a key role in plant defence mechanisms [27]. These compounds are present in foods such as grapes (*Vitis vinifera*), sorghum (*Sorghum bicolor*), and peanuts (*Arachis hypogaea*). Stilbenes have been associated with several health benefits, including cardiovascular protection, anti-obesity and antidiabetic effects, and chemopreventive and neuroprotective properties [28]. Resveratrol, the most well-known stilbene, is particularly abundant in the skin of fresh red grapes [29]. For example, resveratrol (a stilbene) and quercetin (a flavonoid) have been associated with enhanced cardiovascular health [14].

Lignans are naturally occurring polyphenols found in the seeds of oleaginous plants, including flaxseeds, sunflower seeds, linseeds, and sesame seeds [30]. Once ingested, dietary lignans undergo deglycosylation and demethylation by the gut microbiota, leading to the production of bioactive human lignan metabolites such as enterolactone and enterodiol [31]. The potential therapeutic properties of lignans and their derivatives have been reported in cancer chemotherapy, the management of postmenopausal symptoms, and neurodegenerative diseases such as Alzheimer’s disease [32,33]. In addition, lignans have been shown to improve cholesterol regulation, reduce inflammation, and provide antimicrobial and cancer protection [32,34].

Flaxseed-derived lignans, including lariciresinol, pinoresinol, and matairesinol, function as precursors to mammalian phytoestrogens and undergo biotransformation by anaerobic gut bacteria [30]. Through microbial metabolism, these compounds are converted into enterodiol, enterolignans, and enterolactone [35]. Since enterolignans exhibit structural similarities to endogenous oestrogens, this allows them to interact with oestrogen receptors and influence hormone-related physiological processes. This mechanism is thought to contribute to a lower risk of hormone-related cancers, including breast cancer [36,37].

### Underlying Mechanisms of Action

During digestion, flavonoids are metabolised in the intestine, where they are broken down into metabolites before being transported to the liver [38]. Once in the liver, these metabolites enter the enterohepatic circulation via bile excretion, allowing them to reach target cells, be converted into aglycones by the gut microbiota, or be eliminated through urine and faeces [39]. Flavonoid metabolites that pass through the small intestine without being absorbed reach the large intestine, where they can be further degraded by the gut microbiota and subsequently reabsorbed, highlighting the intricate interplay between polyphenols and the gut microbiota [40]. The potential therapeutic effects of dietary polyphenols may be partly explained by their bidirectional interaction with the gut microbiota [41]. Polyphenols influence gut microbiota composition in ways that support overall health, while the gut microbiota, in turn, metabolises polyphenols into bioactive compounds (e.g., phenolic acids) with beneficial physiological effects [42].

Studies have reported that polyphenols modulate inflammation and immune responses through multiple interconnected pathways, including the regulation of Toll-like receptors (TLRs) and NOD-like receptors (NLRs) [38,39,40,41,42]. They also inhibit the NF-κB signalling cascade, modulate inducible enzymes, and alter the expression of pro-inflammatory chemokines, cytokines, and adhesion molecules. For example, resveratrol has been demonstrated to preferentially inhibit lipopolysaccharide (LPS)-induced NF-κB activation by modulating the phosphorylation of IKK and IκB. This inhibition disrupts NF-κB signalling, thereby reducing the production of pro-inflammatory cytokines such as TNF-α and IL-6. As a result, resveratrol exerts potent anti-inflammatory effects [14,29].

Also, polyphenols such as resveratrol activate adenosine monophosphate-activated protein kinase (AMPK), which lowers blood pressure in fructose-induced hypertensive rats [14,29]. It suppresses Rac1-mediated increases in P22Phox subunit activity, reducing oxidative stress. Furthermore, resveratrol enhances the ERK1/2-RSK-nNOS signalling pathway, leading to Ser1416 phosphorylation in nNOS and ultimately promoting nitric oxide (NO) release. This increase in NO contributes to improved endothelial function, reduced oxidative stress, and anti-inflammatory effects. Also, resveratrol mitigates Aβ-induced microglial inflammation, a key driver of neurodegeneration in Alzheimer’s disease, by modulating NLRP3, TLR4/NF-κB, and STAT signalling. By reducing neuroinflammation, resveratrol may help prevent neuronal damage, synaptic dysfunction, and cognitive decline, all hallmarks of dementia [14,29].

## 5. Omega-3 Fatty Acids

Omega-3 fatty acids are essential for cell membrane integrity and function, playing a key role in cellular signalling and physiological regulation. The three main omega-3 fatty acids are alpha-linolenic acid (ALA), eicosapentaenoic acid (EPA), and docosahexaenoic acid (DHA). ALA is predominantly found in chia seeds, walnuts, and plant oils such as flaxseed, canola, and soybean oil [43]. For example, chia seeds, native to South America, are widely recognised for their health-promoting and anti-inflammatory properties, largely due to their high ALA content, which constitutes approximately 70% of their total fat composition [44]. ALA, an essential fatty acid, cannot be synthesised by the human body and must be obtained through dietary intake [45]. While the body has the capacity to convert ALA into EPA and DHA, this process is highly inefficient, with conversion rates typically estimated to be 5–10% and 2–5%, respectively [46]. EPA and DHA are primarily derived from marine sources, including fish and seafood products [47].

Omega-3 fatty acids contribute to blood clotting, inflammation control, and vascular function by serving as precursors to biologically active lipid compounds [48]. A randomised trial by Khalatbari Soltani et al. investigated the effects of flaxseed supplementation in 30 haemodialysis patients with lipid abnormalities. Participants were assigned to either 40 g/day of flaxseed or a control group for eight weeks [49]. The flaxseed group showed significant reductions in C-reactive protein (CRP), total cholesterol, low-density lipoprotein cholesterol (LDL-C), and triglycerides, along with an increase in high-density lipoprotein cholesterol compared to the control group, suggesting potential cardioprotective and anti-inflammatory effects. Similarly, a randomised crossover trial by Rhee and Brunt examined the impact of flaxseed versus wheat bran in nine obese insulin-resistant adults over 12 weeks [50]. While there were no differences in inflammatory markers between both groups, the flaxseed group exhibited a significant decrease in fasting blood glucose compared to the wheat bran group. These study findings suggested that flaxseed supplementation may help improve lipid profiles and glucose metabolism, particularly in individuals with metabolic dysfunction or cardiovascular risk factors. Also, Vuksan et al. conducted a trial involving 77 adults with type 2 diabetes and a BMI between 25 and 40 kg/m^2^, where participants were assigned to consume either chia or a placebo (bran and oats) for six weeks [51]. The chia group showed lower CRP levels, increased adiponectin levels, and significant reductions in both total body weight and waist circumference compared to the placebo. However, further research with larger sample sizes is needed to confirm these effects and determine the optimal dosage for different populations.

Additionally, omega-3 fatty acids influence gene expression through interactions with cell receptors, impacting metabolism and immune responses. Their role in cardiovascular health is well established, with evidence showing that they help lower triglyceride levels, reduce blood pressure, and enhance vascular function [52]. A meta-analysis of 14 randomised controlled trials involving 135,291 participants by Shichun et al. reported that omega-3 supplementation was associated with a significant decrease in major adverse cardiovascular events, myocardial infarction, and cardiovascular mortality [53]. These findings highlight the cardioprotective effects of omega-3 fatty acids in clinical populations.

Due to their anti-inflammatory properties, omega-3 fatty acids are also beneficial in managing autoimmune conditions such as multiple sclerosis, lupus, and rheumatoid arthritis [48]. A randomised controlled trial by Dong et al. demonstrated that participants who took vitamin D3 supplements and marine-derived omega-3 fatty acids over four years experienced a notable decrease in systemic inflammatory markers compared to the placebo group [54]. Another randomised crossover trial by Hutchins et al. investigated the effects of flaxseed supplementation on glycaemic control and inflammation in 25 prediabetic adults [55]. Participants were assigned to one of three interventions for 12 weeks, with either 26 g/day of flaxseed, 13 g/day of flaxseed, or no supplementation (control group). Post-intervention analysis found no significant differences in inflammatory markers between groups. However, fasting blood glucose was significantly lower in participants consuming 13 g of flaxseed compared to the control group. Additionally, insulin levels were significantly reduced in the 13 g/day of flaxseed group compared to both the 26 g/day of flaxseed group and the control group. HOMA-IR, an index of insulin resistance, was significantly lower in the 13 g/day of flaxseed group compared to the 26 g/day of flaxseed group and the control group. The reduction in insulin resistance observed in the flaxseed-supplemented group may be linked to the high fibre content of flaxseed, which is known to slow nutrient absorption, promote short-chain fatty acid (SCFA) production, and support gut microbiota balance [50,56]. However, further research is needed to explore the long-term metabolic effects and optimal dosage of flaxseed supplementation.

Emerging research suggests that omega-3 fatty acids may help reduce cancer risk by regulating cell proliferation and apoptosis [57]. Furthermore, their potential neuroprotective effects are being explored in Alzheimer’s disease, depression, and other neurological disorders [58]. Using the data from the Alzheimer’s Disease Neuroimaging Initiative (ADNI) cohort, which followed 1135 dementia-free participants (mean age = 73 years) for six years., Wei et al. reported that long-term omega-3 supplementation was associated with a 64% lower risk of developing Alzheimer’s disease (hazard ratio: 0.36, 95% confidence interval: 0.18–0.72) [59]. To strengthen these findings, a meta-analysis of 48 cohort studies including 103,651 participants was conducted and reported that higher omega-3 intake was associated with a 20% reduced risk of all-cause dementia or cognitive decline [59]. This effect was particularly pronounced for docosahexaenoic acid (DHA) intake (relative risk [RR]: 0.82, I^2^ = 63.6%, *p* = 0.001) and in studies that adjusted for apolipoprotein APOE ε4 status (RR: 0.83, I^2^ = 65%, *p* = 0.006). Additionally, dose–response analyses demonstrated that each 0.1 g/day increase in DHA or eicosapentaenoic acid (EPA) intake was linked to an 8–9.9% lower risk of cognitive decline (Plinear < 0.0005). Higher levels of plasma EPA (RR: 0.88, I^2^ = 38.1%) and erythrocyte membrane DHA (RR: 0.94, I^2^ = 0.4%) were also associated with a reduced risk of cognitive deterioration [59].

### Underlying Mechanisms of Action

Omega-3 fatty acids are known to suppress the lipogenic gene, enhance lipoprotein lipase expression, promote fatty acid β-oxidation activity, and regulate total body lipid accumulation [60]. On the other hand, omega-6 fatty acids, particularly arachidonic acid (AA), are key precursors for pro-inflammatory eicosanoids, including thromboxanes, leukotrienes (e.g., LTB4), and prostaglandins (e.g., PGE2). However, EPA and DHA compete with AA for incorporation into cell membranes, thereby reducing the substrate availability for AA-derived pro-inflammatory eicosanoid synthesis [43,44,45]. This competitive inhibition shifts the balance towards the reduced production of pro-inflammatory mediators while promoting the generation of anti-inflammatory and pro-resolving metabolites, such as protectins, resolvins, and maresins, which contribute to the resolution of inflammation and overall immune homeostasis [43,44,45,46,47].

In cancer research, omega-3 fatty acids exhibit antineoplastic properties through the activation of the AMPK/SIRT pathway, which is essential for cellular repair and maintenance. This mechanism underlies their potential therapeutic effects in gastric, colorectal, pancreatic, and breast cancers [61]. In the brain, DHA integrates into phospholipid membranes, stabilising and protecting neural structures. This function is particularly relevant in neurodegenerative conditions like Alzheimer’s disease and dementia, where DHA helps preserve neuronal integrity and cognitive function [62,63]. Furthermore, long-chain polyunsaturated fatty acids (PUFAs), including DHA and EPA, contribute to ocular health by targeting pathological processes involved in proliferative and degenerative retinal diseases [64]. Their anti-angiogenic, anti-vaso-proliferative, and neuroprotective properties support retinal cell survival and vascular stability, helping to prevent conditions such as macular degeneration [64].

In the brain, DHA integrates into phospholipid membranes, stabilising and protecting neural structures. This function is particularly relevant in neurodegenerative conditions like Alzheimer’s disease and dementia, where DHA helps preserve neuronal integrity and cognitive function [62,63]. In Alzheimer’s disease, Aβ is derived from the amyloid precursor protein (APP). When APP is cleaved by α-secretases within the Aβ domain, this generates α-secretase-cleaved soluble APP (sAPPα), which exhibits neurotrophic and neuroprotective properties. EPA and DHA enhance membrane fluidity, which in turn promotes the secretion of α-secretase-cleaved sAPPα. This process reduces Aβ formation and supports neuronal health, suggesting a potential protective role of omega-3 fatty acids in Alzheimer’s disease [62,63].

## 6. Probiotics

Probiotics consist of beneficial microorganisms that support gut health and overall well-being [65]. Most probiotic strains belong to lactic acid bacteria, including *Bifidobacterium*, *Lactobacillus*, *Streptococcus*, *Lactococcus*, and *Enterococcus*. Additionally, certain yeast species, particularly those from the genus *Saccharomyces*, are recognised for their probiotic properties [66]. The primary dietary sources of probiotics are fermented foods, which naturally contain these beneficial microbes. Common probiotic-rich foods include yoghurt, sauerkraut, kefir, and kimchi. The regular consumption of these foods has been associated with improved digestion, enhanced immune function, and potential benefits in metabolic and inflammatory disorders [67]. Therefore, the therapeutic potential of probiotics and their role in modulating the gut–brain axis, improving metabolic health, and reducing inflammation has become an area of growing interest.

Probiotic consumption allows beneficial microorganisms to colonise the gastrointestinal tract, where they play a key role in maintaining gut homeostasis. Probiotics are particularly effective in the management of various types of diarrhoea, including antibiotic-associated diarrhoea in adults [68]. In addition, they have been found to alleviate symptoms of inflammatory bowel disease by modulating gut microbiota composition and immune responses [69].

Probiotics have broader therapeutic potential beyond gut health, with evidence suggesting their role in the prevention and treatment of allergic diseases, high cholesterol, atopic dermatitis, and cancer [70,71,72]. Their health benefits are largely attributed to the competitive inhibition of pathogenic microorganisms, antimicrobial activity against harmful bacteria, modulation of the gut microbiota, and regulation of the host immune response. Recent studies reported that next-generation probiotics, derived from next-generation microorganisms that are isolated using advanced techniques, may offer enhanced therapeutic potential [73]. For example, *Christensenella minuta* and *Prevotella copri* have been implicated in modulating insulin resistance [74,75]. *Bacteroides fragilis* has demonstrated anti-cancer properties and anti-inflammatory effects, while *Faecalibacterium prausnitzii* has been shown to protect against intestinal diseases in animal models [76,77].

Probiotic supplementation, either alone or in combination with selenium or vitamin D, has been shown to significantly improve mental health biomarkers and metabolic profiles in diabetic patients with coronary heart disease [78,79]. These benefits include reductions in levels of high-sensitivity C-reactive protein (hs-CRP), LDL, total cholesterol, nitric oxide (NO), markers of inflammation, and oxidative stress. Increasing evidence suggests that gut microbiota alterations play a critical role in the development of atherosclerosis, a major risk factor for coronary heart disease and stroke. These microbial changes influence cholesterol and uric acid metabolism, oxidative stress, and inflammatory pathways through the production of various metabolites. A whole-genome sequencing study by Karlsson et al. identified a potential link between gut microbiota composition and atherosclerotic heart disease [80]. Specifically, patients with atherosclerosis exhibited a higher abundance of *Collinsella* and a reduced presence of *Rothia* and *Eubacterium* spp. compared to healthy controls. Additionally, individuals with coronary heart disease showed a significant reduction in the *Firmicutes*-to-*Bacteroidetes* (F/B) ratio, a key indicator of gut dysbiosis. In ischemic stroke patients, microbial diversity was reduced, while the abundance of *Bacteroidetes* was increased [81]. Two studies by Emoto et al. revealed distinct microbial shifts in patients with coronary heart disease, including an increased abundance of *Lactobacillus* species and a notable reduction in *Bacteroidetes*, including *Bifidobacterium* and *Prevotella*, compared to healthy individuals [82,83]. In the context of stroke, significant alterations in gut microbiota composition have also been observed. Post-stroke microbial changes include an increased abundance of *Clostridial* species and *Akkermansia muciniphila* [84].

Studies investigating synbiotic supplementation (probiotics with the prebiotic inulin) in patients with chronic kidney disease and coronary heart disease reported improvements in several cardiometabolic biomarkers, including cholesterol, NO, hs-CRP, and malondialdehyde (MDA) (a marker of oxidative damage) [85,86]. *Lactobacillus plantarum 299v* (*Lp299v*) supplementation improved vascular endothelial function and reduced systemic inflammation in men with coronary artery disease [87]. The co-supplementation of probiotics and inulin for eight weeks in patients with coronary artery disease had positive effects on anxiety, depression, and inflammatory markers [88]. Also, *Lactobacillus rhamnosus GG* supplementation was associated with a reduction in metabolic endotoxemia and systemic inflammation in patients with coronary artery disease [89]. These probiotic strains have demonstrated anti-inflammatory effects by reducing the levels of pro-inflammatory cytokines, including IL-8, IL-12, IL-1β, and tumour necrosis factor-alpha (TNF-α). Also, probiotics may have neuroprotective effects in stroke prevention. The induction of probiotic strains, including *Bifidobacterium breve*, *L. casei*, *L. bulgaricus*, *Clostridium butyricum*, *Bacillus licheniformis*, and *L. acidophilus*, has been linked to reduced inflammatory cytokine levels and improved stroke outcomes in animal models [90,91,92].

Probiotics exert immunomodulatory effects by stimulating both the innate and adaptive immune systems, thereby contributing to host health. Specific strains such as *Lactobacillus paracasei CNCM I-1518* and *Lactobacillus casei CRL 431* interact with intestinal epithelial cells via Toll-like receptors, modulating immune responses by increasing the production of macrophage chemoattractant protein-1 (MCP1) and interleukin-6 (IL-6) [93,94]. Probiotics also play a role in strengthening the intestinal barrier, a key defence mechanism against pathogens. They contribute to mucus layer reinforcement by increasing the number of Goblet cells and upregulating mucin-related proteins (MUC2, MUC3, and MUC5AC) in intestinal cells (HT29) [95].

Probiotics also influence the gut microbiota composition, altering specific commensal bacteria such as *Oscillibacter* and *Prevotella*. These microbes produce anti-inflammatory metabolites that promote the differentiation of anti-inflammatory Treg and Type 1 regulatory T (Tr1) cells and reduce Th17 cell polarisation, further reinforcing immune balance and systemic anti-inflammatory effects [96].

### Underlying Mechanisms of Action

Four different mechanisms by which probiotics exert beneficial effects on human health have been proposed as follows: (1) probiotics help strengthen the intestinal barrier, reducing permeability and preventing the translocation of harmful pathogens and toxins; (2) beneficial microbes outcompete harmful bacteria by depriving them of essential nutrients and occupying adhesion sites on the intestinal mucosa; (3) probiotics influence immune responses and neurotransmitter synthesis, impacting gut–brain communication and overall physiological function; and (4) probiotics regulate immune system activity, reducing excessive inflammation and promoting immune homeostasis [97]. For example, stroke patients reported that they experienced gastrointestinal complications, including gut dysmotility, dysbiosis, and increased intestinal permeability (leaky gut), which were associated with poorer stroke outcomes [98]. One proposed explanation for the underlying mechanism is the gut–brain axis. Sympathetic nervous system (SNS) activation plays a key role in inflammation-induced vascular endothelial dysfunction and cardiometabolic disease. Since some gut bacteria, such as *Lactobacillus* and *Bifidobacterium*, are capable of producing neurotransmitters including serotonin, γ-aminobutyric acid (GABA), norepinephrine, and dopamine, the alterations in gut microbiota composition via probiotic supplementation could influence neurotransmitter levels. This, in turn, may modulate SNS activity and impact cardiometabolic functions [99].

Therefore, the primary mechanism by which probiotics exert these benefits is through counteracting pathogenic microbes while promoting a balanced gut microbiota. By enhancing the growth of beneficial bacteria and inhibiting harmful pathogens, probiotics help restore microbial equilibrium, which is essential for digestive health, immune function, and overall gastrointestinal integrity [100]. Oral probiotic administration has been shown to increase Paneth cell numbers and enhance tight junction signalling, further supporting gut barrier integrity and anti-inflammatory responses [101].

Probiotics modulate the gut–brain-microbiota axis by mitigating gut dysbiosis-induced inflammation and preventing the hyperactivation of the hypothalamic–pituitary–adrenal axis. For example, *Bifidobacterium* and *Lactobacillus* species enhance synaptic plasticity by normalising long-term potentiation, thereby improving neuronal signal transmission in the brain. This effect is accompanied by a significant reduction in microglial activation markers and an increase in the brain-derived neurotrophic factor and synapsin expression, both essential for neuronal survival, synaptic function, and neurogenesis. These changes correlate with improved cognition and spatial learning, suggesting a potential neuroprotective role of probiotics in neurodegenerative and cognitive disorders. In addition, probiotics enhance Sirtuin-1 (SIRT1), a NAD^+^-dependent deacetylase that reduces ROS levels, promotes neuronal survival, and inhibits apoptosis via PARP deacetylation. They also upregulate RARβ, facilitating non-amyloidogenic APP processing by activating ADAM10 (α-secretase) through SIRT1-mediated deacetylation [99,100]. This prevents Aβ peptide formation and deposition, potentially protecting against Alzheimer’s disease.

## 7. Prebiotics

Prebiotics are defined by specific selection criteria that determine their ability to support gut health and selectively stimulate beneficial gut microbiota. Some of these criteria include the selective promotion of probiotic growth; selective fermentation by beneficial microbiota; positive effects on host health; stability during food and feed processing; and resistance to digestion in the upper gastrointestinal tract [102]. Some of the common prebiotics include oligosaccharides (particularly fructooligosaccharides (FOSs) and inulin) and galactooligosaccharides (GOSs) [103]. Epidemiological and clinical studies indicate that diets high in fibre and prebiotics are associated with a lower risk of type 2 diabetes, a decreased prevalence of colorectal cancer, and reduced body weight [104].

Prebiotic supplementation has been shown to exert beneficial effects on cardiovascular health by improving lipid profiles, reducing inflammation, enhancing antioxidant capacity, and restoring gut microbiota balance [104]. Studies indicate that inulin and inulin-based co-supplementation can lower cholesterol, total cholesterol, LDL, CRP, and inflammatory cytokines while also mitigating gut microbiota dysbiosis [104]. Additionally, inulin has been associated with improved antioxidant parameters, which may contribute to its cardioprotective effects [104].

Other prebiotics have demonstrated cardiovascular benefits. Dietary supplementation with soluble fibre (Minolest) improved lipid profiles in individuals with mild hypercholesterolemia and a low risk of coronary artery disease [105]. Chitosan oligosaccharides (COSs) are reported to exhibit cardioprotective effects in coronary heart disease by improving lipid metabolism and enhancing antioxidant defences through the modulation of gut microbiota [106]. A fermented wheat bran-based prebiotic complex was found to correct gut dysbiosis and reduce endotoxemia in female rats with heart failure [107]. In a rat model of ischemia–reperfusion, larch arabinogalactan (an active component of pectin) was shown to attenuate myocardial injury by inhibiting apoptotic pathways [108].

Short-chain fatty acids (SCFAs) such as acetate, butyrate, and propionate are the primary metabolites produced through prebiotic fermentation by gut bacteria. These bioactive compounds exert numerous health benefits including enhancing intestinal barrier integrity, reducing body weight, and improving glycaemic control [109]. SCFAs exert their effects by binding to G-protein-coupled receptors (GPCRs), such as free fatty acid receptors (FFAR) 2 and 3 and olfactory receptor 78 (Olfr78), which primarily interact with acetate and propionate, and GPR109A, which exhibits a higher affinity for butyrate [110]. In addition, SCFAs act as histone deacetylase inhibitors (HDACs), influencing chromatin structure and gene expression to regulate key metabolic and inflammatory pathways [111].

### Underlying Mechanisms of Action

Prebiotics exert beneficial effects on host health through several mechanisms, primarily by influencing gut microbiota composition, the gut environment, and immune function. Prebiotics, whether naturally present in food or added as supplements, serve as substrates for fermentation by gut bacteria. This fermentation process leads to alterations in gut microbial composition, promoting the growth of beneficial bacteria (e.g., *Bifidobacterium* and *Lactobacillus*) while suppressing potential pathogens. Additionally, prebiotic fermentation produces SCFAs, which contribute to metabolic regulation and gut homeostasis. In addition, the production of SCFAs from prebiotic fermentation lowers colonic pH, further inhibiting the growth of harmful microbes and improving nutrient absorption and gut barrier function [94].

Therefore, prebiotics play a crucial role in modulating the immune response and enhancing host defences by influencing both innate and adaptive immunity. Different types of prebiotics exert varied immunological effects, with dendritic cells (DCs) being the most reported immune cells affected by prebiotic supplementation [94]. Among them, butyrate is the most effective in modulating immune responses, particularly in the regulation of dendritic cells and T-cell function [112]. Beyond immune regulation, SCFAs influence brain function and host physiology through the gut–brain axis. These microbially derived metabolites are involved in neurotransmission and nerve activation, which impact cognitive and emotional processes [113,114].

In obesity and insulin resistance, hyperglycaemia can increase gut permeability, triggering an inflammatory cascade that worsens metabolic dysfunction [115]. SCFAs play a critical role in maintaining gut epithelial integrity by regulating tight junction proteins. Butyrate enhances tight junction stability through nucleotide-binding oligomerisation domain-like receptors (NLRs), which subsequently modulate inflammation [116]. SCFAs also influence appetite control and energy balance, thereby helping protect against obesity [117]. They contribute to glucose homeostasis by improving insulin sensitivity, strengthening the gut barrier, and enhancing anti-inflammatory and antioxidant defences [118].

In Alzheimer’s disease models, lactulose, the first commercially available prebiotic and a common food additive, has demonstrated cognitive benefits by alleviating short-term memory impairment and learning deficits, suggesting a potential role in neuroprotection through gut–brain axis modulation. In addition, fructooligosaccharides have been shown to restore gut microbiota balance, mitigate cognitive deficits, and reduce neuropathological changes in APP/PS1 transgenic mice. These effects are mediated via the gut microbiota-GLP-1/GLP-1R pathway, leading to increased synapsin I expression and reduced JNK phosphorylation, both of which are critical for synaptic function and neuroprotection [119,120].

## 8. Summary

The growing body of evidence has highlighted the modulatory role of bioactive compounds in functional foods to prevent chronic diseases and improve metabolic functions. Polyphenols, omega-3 fatty acids, prebiotics, and probiotics have demonstrated cardioprotective, neuroprotective, anti-inflammatory, and gut-modulating properties, making them essential components of a health-promoting diet. The fermentation of prebiotics into SCFAs further underscores their role in enhancing gut barrier integrity, regulating blood pressure, and improving insulin sensitivity. Despite the promising findings, further research is needed to refine the understanding of optimal dosages and the long-term clinical implications of functional foods. In addition, future studies should explore personalised nutrition approaches, considering individual microbiota composition, genetic predisposition, and lifestyle factors.

## Figures and Tables

**Table 1:** Definitions of functional foods.

Source	Definition
World Health Organization (WHO)	Contain bioactive compounds that influence health, disease prevention, and overall physiological function.
U.S. Food and Drug Administration	Not legally defined but are recognised as foods that provide health benefits beyond nutrient content.
European Commission	Must demonstrate scientifically proven benefits in improving health and reducing disease risk when consumed as part of a normal diet.
Japanese Ministry of Health, Labour and Welfare (FOSHU)	Foods with approved health claims, scientifically validated to provide specific health benefits beyond basic nutrition.
Academy of Nutrition and Dietetics	Whole foods and fortified, enriched, or enhanced foods that provide health benefits beyond basic nutrition.
Institute of Food Technologists (IFT)	Conventionally or modified foods that provide bioactive compounds beneficial to health and well-being.
International Life Sciences Institute (ILSI)	Offer physiological benefits or reduce the risk of chronic disease beyond their basic nutritional functions.

**Table 2:** Examples of commonly consumed functional foods and their bioactive properties.

Functional Food	Bioactive Compounds	Health Benefits
Flaxseeds and chia seeds	ALA, lignans	Cardiovascular health, anti-inflammatory, hormone balance
Yoghurt and kefir	Probiotics (*Lactobacillus*, *Bifidobacterium*)	Gut health, immune modulation, improved digestion
Fermented foods (e.g., kimchi, miso, tempeh)	Probiotics	Gut microbiota balance, immune health, anti-inflammatory
Berries (blueberries, strawberries, raspberries)	Polyphenols (anthocyanins, flavonoids)	Antioxidant, anti-inflammatory, cognitive benefits
Broccoli and cruciferous vegetables	Sulforaphane, glucosinolates	Anti-cancer, detoxification, anti-inflammatory
Dark chocolate	Flavonoids, polyphenols	Cardiovascular protection, mood enhancement, antioxidant
Nuts (e.g., walnuts, almonds, pistachios)	Polyphenols, omega-3 fatty acids, tocopherols	Heart health, brain function, anti-inflammatory
Legumes (lentils, chickpeas, beans)	Fibre, polyphenols, resistant starch	Blood sugar control, gut health, reduces cholesterol
Citrus fruits (oranges, lemons, grapefruits)	Vitamin C, flavonoids	Immune support, skin health, antioxidant

**Table 3:** Examples of polyphenols and their food sources.

Polyphenols	Some Food Sources (Refs. [11,12,13,14,15,16,17,18,19,20,21,22,23,24,25,26,27,28,29,30])
*(a) Flavonoids*	
Apigenin	Celery
Quercetin	Pomegranate
Naringenins	Orange
Cyanidin	Blueberries
Catechin	Tea
*(b) Phenolic acids*	
Ferulic acid	Cereals
Gallic acid	Fruit and vegetables
Chlorogenic acid	
Rosmarinic acid	Rosemary
Caffeic acid	Coffee
*(c) Lignans*	
Podophyllotoxine	Rye
*(d) Stilbenes*	
Resveratrol	Grapes

## Data Availability

No new data were created or analysed in this study.
